# Supplementary figures and images for: Meta-Analysis of Microarray Data and Their Utility in Dissecting the Mapped QTLs for Heat Acclimation in Rice
Source: Plants (Basel). 2023 Apr 18;12(8):1697. doi: 10.3390/plants12081697 (PMC10142300; doi:10.3390/plants12081697)

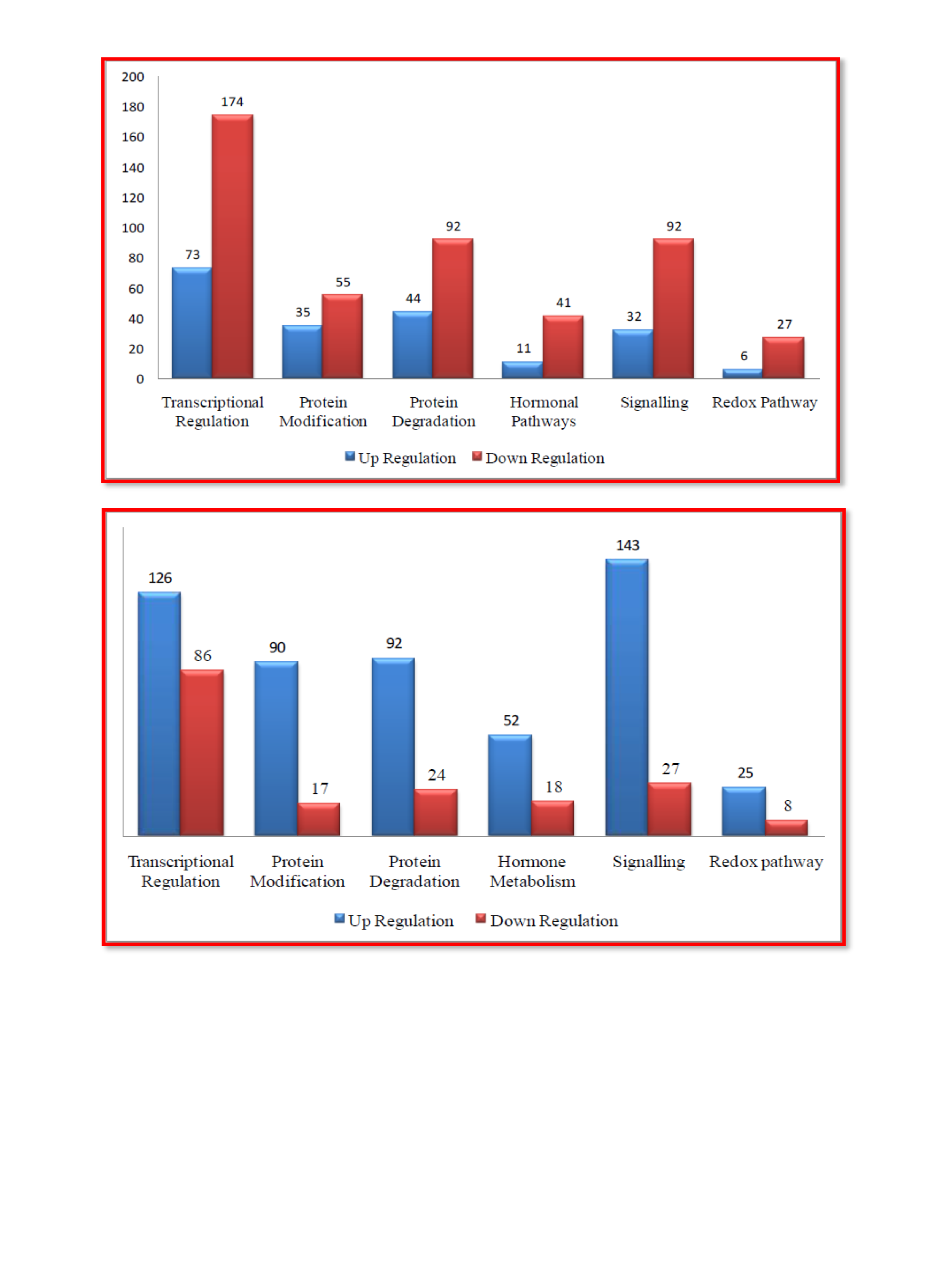

Supplement: Supplementary file 1 [file plants-12-01697-s001.zip › Supplementary Figure S1.tif]

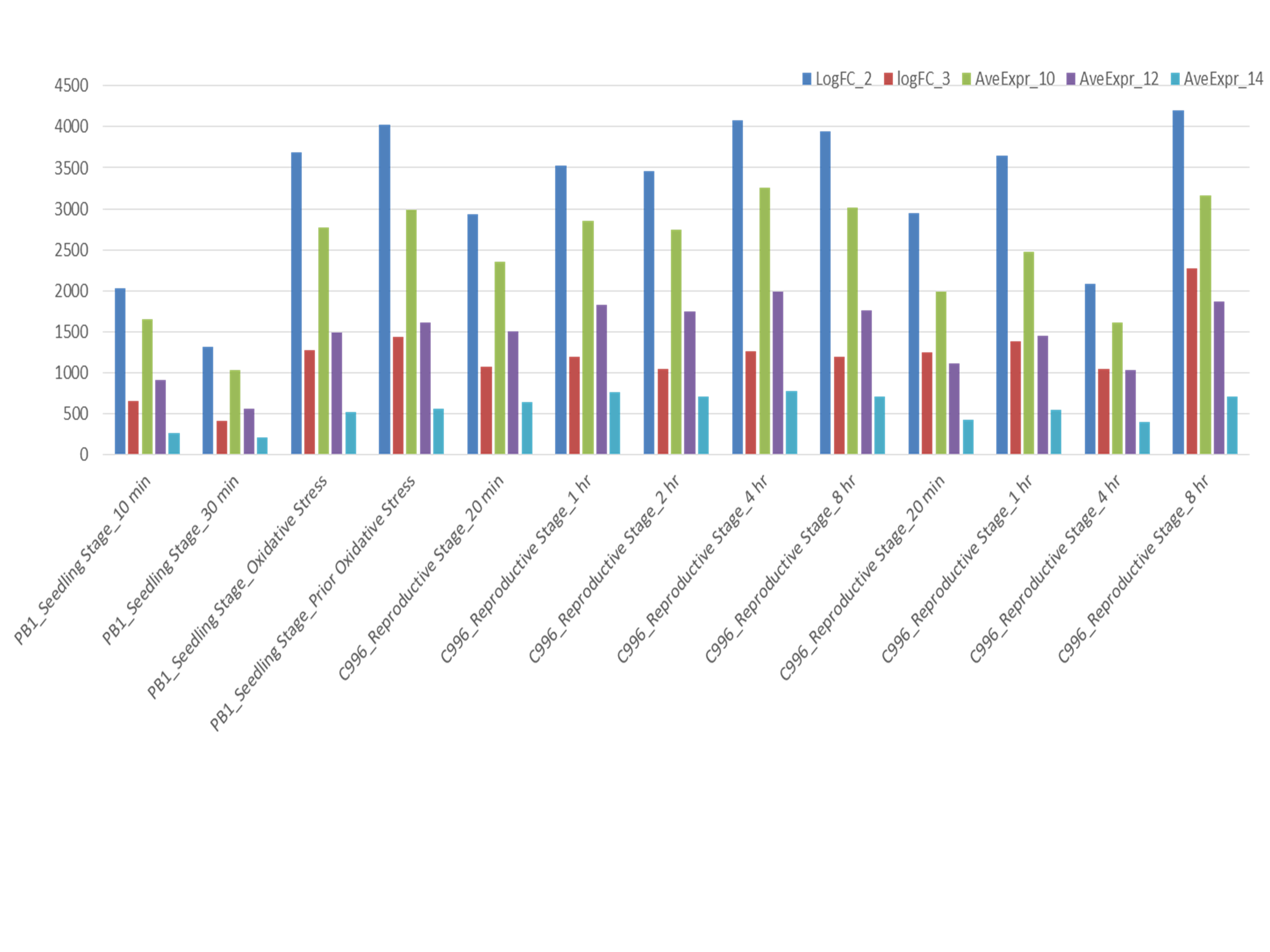

Supplement: Supplementary file 1 [file plants-12-01697-s001.zip › Supplementary Figure S2.tif]

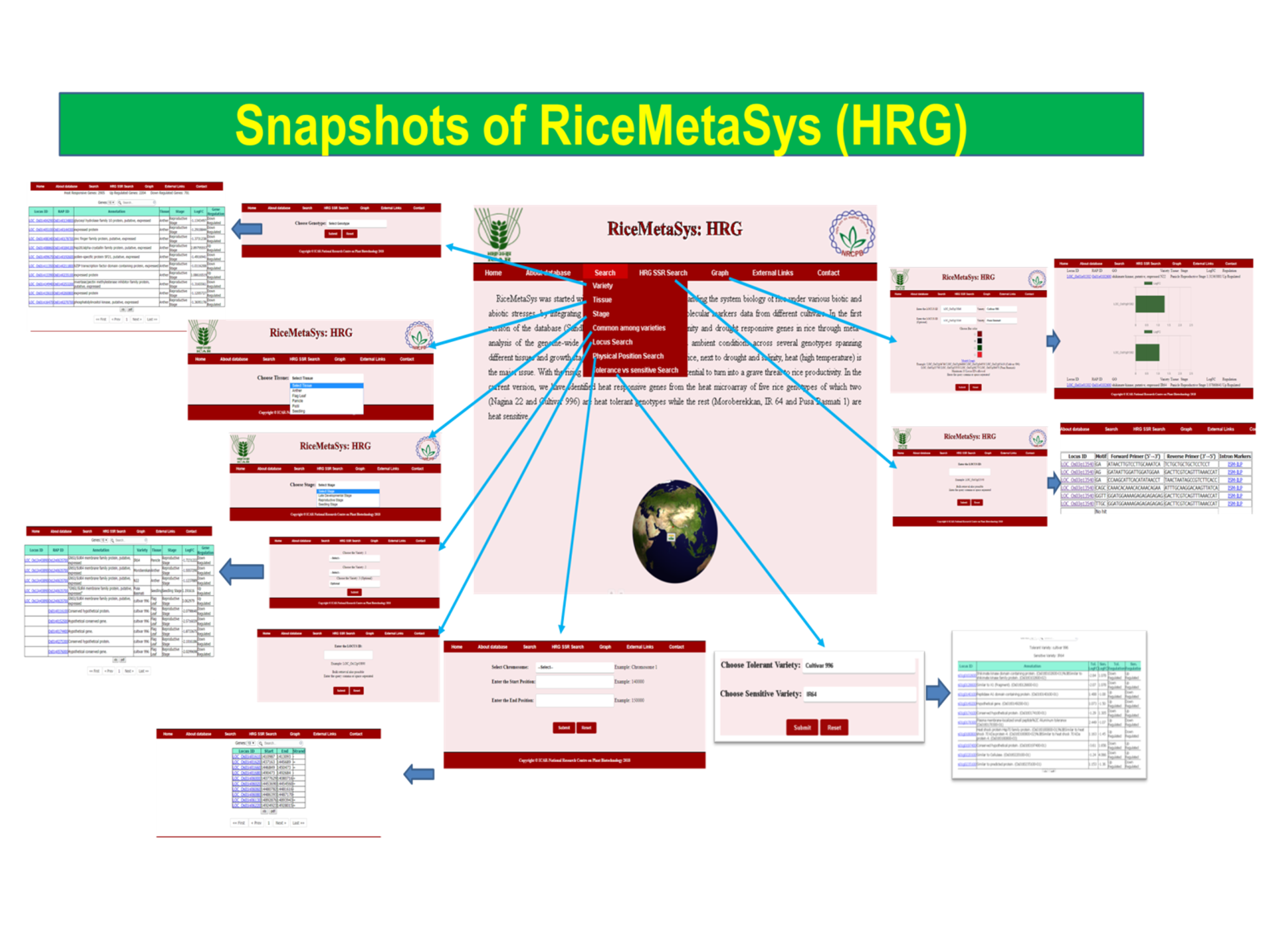

Supplement: Supplementary file 1 [file plants-12-01697-s001.zip › Supplementary Figure S3.tif]

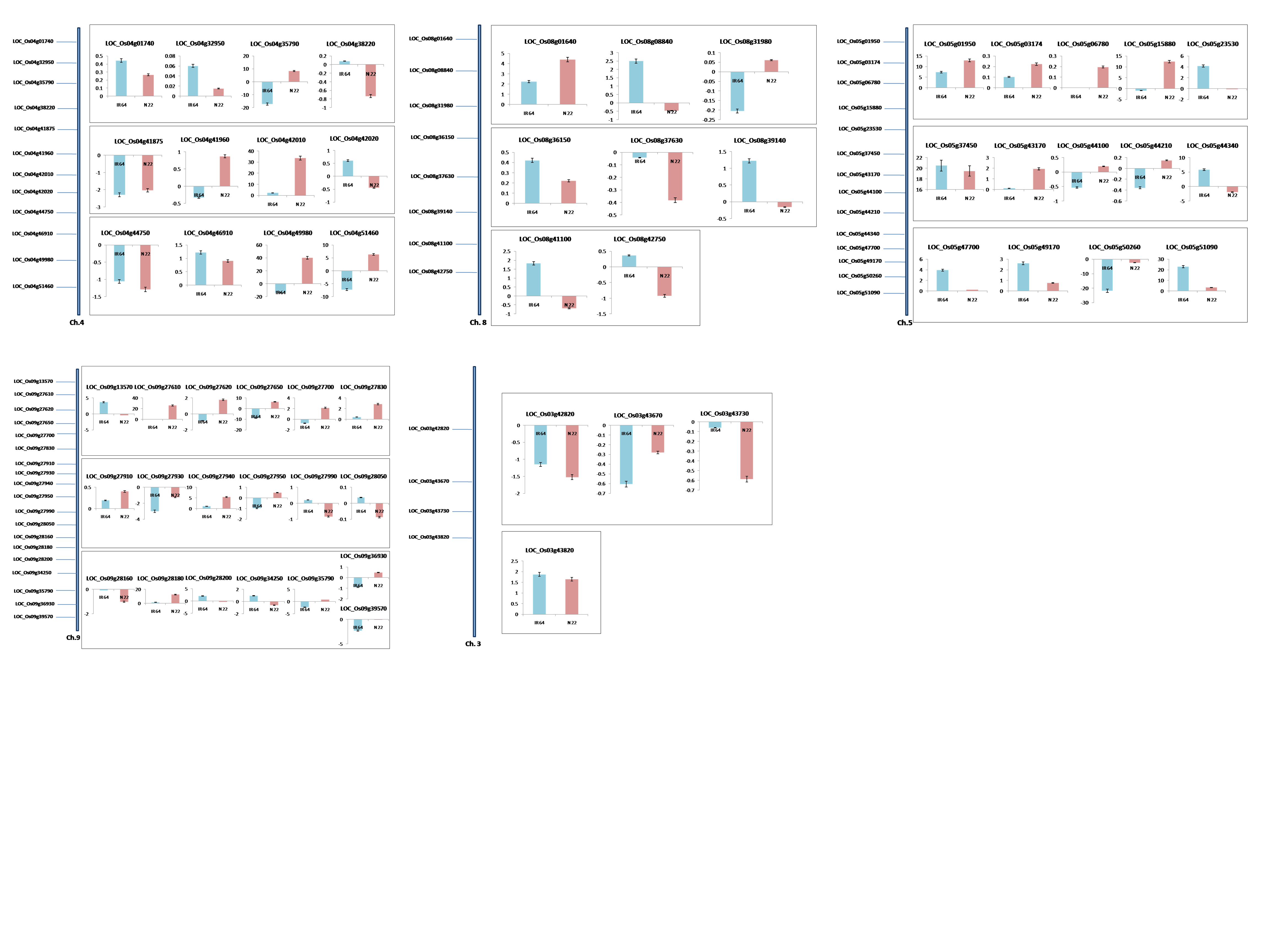

Supplement: Supplementary file 1 [file plants-12-01697-s001.zip › Supplementary Figure S4.tif]
